# Detection method of viral pneumonia imaging features based on CT scan images in COVID-19 case study

**DOI:** 10.1016/j.mex.2023.102507

**Published:** 2023-12-19

**Authors:** Fajar Astuti Hermawati, Bambang Riyanto Trilaksono, Anto Satriyo Nugroho, Elly Matul Imah, Lukas, Telly Kamelia, Tati L.E.R. Mengko, Astri Handayani, Stefanus Eric Sugijono, Benny Zulkarnaien, Rahmi Afifi, Dimas Bintang Kusumawardhana

**Affiliations:** a Department of Informatics, Universitas 17 Agustus 1945, Surabaya, Indonesia; b School of Electrical Engineering and Informatics, Institut Teknologi Bandung, Bandung, Indonesia; c National Research and Innovation Agency, Indonesia; d Data Science Department, Universitas Negeri Surabaya, Indonesia; e Electrial Engineering Department, Universitas Katolik Indonesia Atma Jaya, Jakarta, Indonesia; f Department of Internal Medicine, Dr. Cipto Mangunkusumo National Central Public Hospital, Jakarta, Indonesia; g Department of Radiology, Eka Hospital Bekasi, Indonesia; h Department of Radiology, Dr. Cipto Mangunkusumo National Central Public Hospital, Jakarta, Indonesia

**Keywords:** Image segmentation, Image thresholding, Ground-Glass-Opacity, Consolidation, Severity level, Morphological image processing & image thresholding

## Abstract

This study aims to automatically analyze and extract abnormalities in the lung field due to Coronavirus Disease 2019 (COVID-19). Types of abnormalities that can be detected are Ground Glass Opacity (GGO) and consolidation. The proposed method can also identify the location of the abnormality in the lung field, that is, the central and peripheral lung area. The location and type of these abnormalities affect the severity and confidence level of a patient suffering from COVID-19. The detection results using the proposed method are compared with the results of manual detection by radiologists. From the experimental results, the proposed system can provide an average error of 0.059 for the severity score and 0.069 for the confidence level. This method has been implemented in a web-based application for general users.

• A method to detect the appearance of viral pneumonia imaging features, namely Ground Glass Opacity (GGO) and consolidation on the chest Computed Tomography (CT) scan images.
• This method can separate the lung field to the right lung and the left lung, and it also can identify the detected imaging feature's location in the central or peripheral of the lung field.
• Severity level and confidence level of the patient's suffering are measured.

## Graphical abstract

**Figure:**
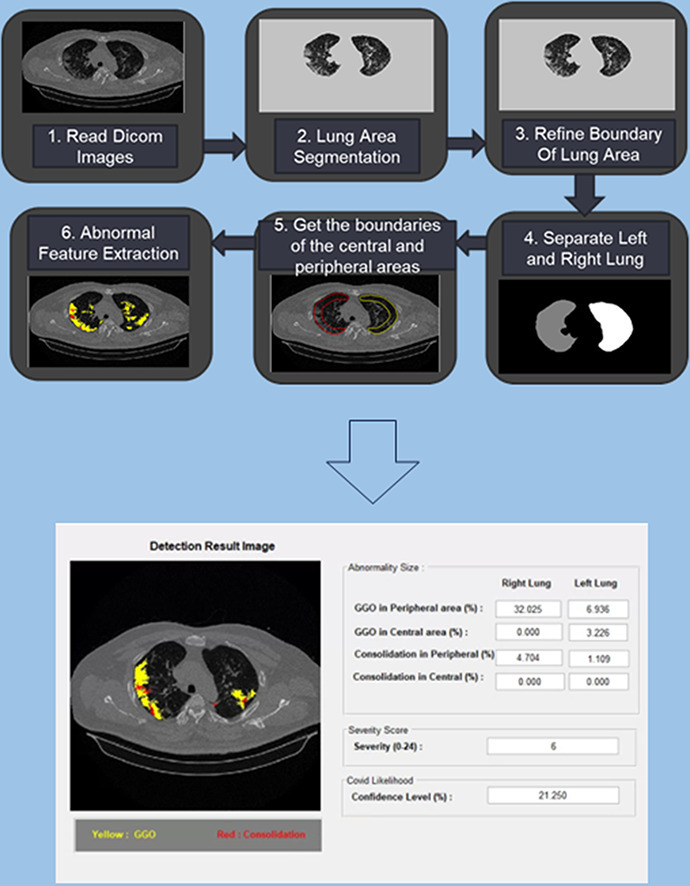

Specifications table

**Table:** 

Subject area:	Computer Science
More specific subject area:	Medical image processing
Name of your method:	Morphological image processing & image thresholding
Name and reference of original method:	N/A
Resource availability:	Application: https://platform.tfric-19.id/ Data will be made available on request.

**Method details**

## Introduction

Viral pneumonia is a challenging condition as its diagnosis often proves difficult and commonly relies on ruling out bacterial causes. Treatment inefficacy results from the limited availability of effective molecules targeting the viruses typically implicated in the illness [1]. COVID-19, also referred to as Coronavirus disease 2019, is an infective ailment prompted by the Coronavirus. This virus leads to acute respiratory syndrome 2 (SARS-CoV-2), which was formerly labeled as 2019 novel coronavirus (2019-nCoV), belonging to the Coronavirus family. The initial instances were identified in December 2019 in Wuhan, China, and subsequently disseminated worldwide. On the 11th of March 2020, the World Health Organization officially designated the ongoing outbreak as a pandemic [2], [3], [4], [5]. COVID-19 displays clinical features that are infrequently observed in cases of pneumonia caused by other viruses. The general symptoms related to lung involvement are often similar and difficult to distinguish from those seen in other types of viral pneumonia [6].

Numerous radiology associations have declared that CT scans ought not to be employed as the foremost means of diagnosis or screening for COVID-19. On March 16, 2020, a group of experts in Singapore communicated that the outcomes of CT scans did not align with the established diagnostic criteria for COVID-19. Nevertheless, CT scan findings have been adopted as an alternative diagnostic examination [7,8]. The main observation associated with COVID-19 is a viral pneumonia that appears either atypical or organized. In instances where the disease is in its early or mild stages, as many as 18% of cases exhibit a normal chest X-ray or CT scan. Involvement affecting both lungs and/or multiple lobes is frequently observed. Examination utilizing standard X-ray imaging has indicated irregular or hazy patches within the airspaces. In adults, the predominant CT findings typically consist of ground-glass opacity (GGO), consolidation of air spaces, a pattern resembling crazy paving (involving GGOs and thickening of inter- and intra-lobular septa), thickening of broncho-vascular structures within the affected area, and the presence of traction bronchiectasis [9], [10], [11], [12], [13], [14], [15], [16].

Ground-glass opacity and/or consolidation of air spaces typically manifest bilaterally in a peripheral and basal manner. In a retrospective analysis of 112 patients, it was observed that 54% of individuals without symptoms displayed alterations in lung images on CT scans [12,15,17]. A research paper released in March 2020 [18] assessed the capability of Chinese and American radiologists to differentiate COVID-19 from other forms of viral pneumonia *via* CT scans. Chinese radiologists exhibited a sensitivity ranging from 72% to 94% and a specificity between 24% and 94%. Contrastingly, US radiologists achieved superior outcomes, boasting a 100% specificity rate for two of the radiologists. It is important to note, however, that the American experts worked with a considerably smaller dataset compared to their Chinese counterparts. Within the confines of this study, the chest CT discoveries that held the utmost discriminatory significance (*p* < 0.001) encompassed peripheral distribution, opacity, and thickening of bronchial vasculature within the lesion.

The example of a CT scan image from a positive COVID-19 patient in Italy shows a reasonably wide fog/gray area on the left and right. GGO is a prevalent abnormality found on chest CT, not just COVID-19. But there are some features that, when they appear together, indicate viral pneumonia (can be influenza, COVID-19, or other coronaviruses). So, the pattern suggests viral pneumonia. It is difficult to interpret in the early phase when the number of GGOs is small (maybe only 1). When the stage is a bit more advanced, the pattern has already been formed; it is more indicative. On the other hand, other abnormalities may appear in co-morbidities that disguise the picture of viral pneumonia, which can lead to false negatives. So it's not easy either; it really depends on many other factors [19].

Several studies to detect the imaging features of COVID-19 patients utilize a deep learning approach [19], [20], [21], [22], [23], [24], [25], which requires enormous amounts of data. Li et al. [19] implemented a model called COVID-19 detection neural network (COVNet) to extract visual characteristics from detailed chest CT scans to identify instances of COVID-19. The COVNet model was tested using CT scans depicting community-acquired pneumonia (CAP) and various non-pneumonia anomalies. Ennab and Mcheick [20] utilized an interpretability-focused model to analyze and understand the forecasts made by the CNN model concerning COVID-19 patients, based on their chest CT scans. In Ref. [21] research, five pre-existing convolutional neural network models (ResNet50, ResNet101, ResNet152, InceptionV3, and Inception-ResNetV2) were suggested for identifying patients with coronavirus pneumonia through chest X-ray images. Upon analyzing the performance outcomes, it was evident that among these models, the pre-trained ResNet50 model demonstrated the most effective classification performance. In Ref. [22] study, several CNN models are used to classify a dataset of CT images and calculate the likelihood of COVID-19 infection. The areas potentially affected by an infection were delineated from the pulmonary CT image collection utilizing a 3D deep learning model. Wang et al. [23] adapted the inception transfer-learning model to create the algorithm, conducting subsequent internal and external validation. Transfer learning was executed by training with a predefined model, specifically utilizing the widely recognized GoogleNet Inception v3 CNN. Suri et al. [24] proposed automated lung segmentation using a hybrid deep learning ResNet-UNet model, incorporating automatic adjustment of Hounsfield units, hyperparameter optimization, and training conducted in parallel and distributed setups. Furtado et al. [25] introduced a 3D VGG-based CNN structure designed to precisely diagnose COVID-19 from chest CT scans. This 3D model can detect connections among neighboring slices, a capability beyond the reach of 2D networks that rely solely on spatial voxel data within individual slices.

While these DL methods showcase significant advancements in AI-based systems, their scope is constrained due to the absence of ground truth (GT) lung ROI segmentations. Manual annotation and segmentation, time-consuming tasks requiring expertise, often introduce subjectivity influenced by individual methodologies. Consequently, the assessment of COVID-19 CT findings has primarily been confined to qualitative or semi-quantitative evaluations. Furthermore, DL algorithms are frequently trained on datasets derived from identical CT machines and annotated by the same set of radiologists. During the pandemic, obtaining annotated CT scan images of COVID-19 patients in Indonesia from radiology experts has been very challenging. Apart from policy constraints limiting activities, hospitals are also extremely busy due to the increasing number of patients requiring care. Here is the reason why we employ a non-deep learning approach to detect imaging features in COVID-19 patients based on CT scan images. Few studies on COVID-19 detection on CT scan images apply image processing approaches, such as thresholding [26,27] and morphological operation [28]. Yousef et al. [26] employed the density-based multi-level thresholding technique to measure COVID-19 lung involvement in high-resolution, thin-cut volumetric CT images of the entire lung. Khan et al. [27] introduced a semi-automated segmentation technique using thresholds to create infection-specific region of interest (ROI) delineations on lung CT scans. These infection masks are subsequently employed to compute the percentage of lung abnormality (PLA), gauging COVID-19 severity and examining disease progression in follow-up CT scans. In Ref. [28], a morphological reconstruction process is initially applied to eliminate external disturbances affecting infected regions and precisely identify areas of interest. The Edge Content-based contrast matrix method determines the optimal structuring element size. Following this, the morphological reconstruction operation is used for opening to remove noise further, and subsequently, closing-based reconstruction aids in noise reduction.

Our research proposes a technique that combine the thresholding approach and morphological operation for identifying visual signs of viral pneumonia, specifically Ground Glass Opacity (GGO) and consolidation, on chest CT scan images. This approach enables the segmentation of the lung field into right and left lobes, determining the location of identified imaging features as either central or peripheral within the lung field. It assesses the severity level and confidence of the patient's condition. Our method has been implemented and can be accessed at https://platform.tfric-19.id/.

## Proposed methods

### CT imaging features of COVID-19

SARS-CoV-2, an RNA virus with a single strand, enters human cells through the angiotensin-converting enzyme II (ACE2), causing damage to the pulmonary interstitium and parenchyma. Recent findings indicate that diverse features in chest CT scans correspond to different stages and severity of the disease in patients infected with SARS-CoV-2 [29]. Throughout the progression of COVID-19, distinct CT characteristics are observed, suggesting that multiple CT scans can be beneficial in tracking the disease's development and facilitating prompt treatment. A key feature of COVID-19 is the appearance of scattered ground glass opacities (GGOs) on both sides of the lungs, which may merge into concentrated, solidified areas. These typically manifest in the outer regions near the pleura and adjacent to the bronchovascular bundles [30]. Ground glass opacity (GGO) and consolidation represent the predominant CT indications of COVID-19 pneumonia [29], [30], [31], [32], [33], [34], [35], [36], [37].

GGOs are non-specific observations described as cloudy lung opacities that do not mask the underlying vascular or bronchial boundaries. They are believed to be connected to either a partial filling of airspaces or thickening in the interstitial areas [30]. Consolidations result from the total substitution of alveolar airspaces by abnormal fluids or cells, causing an elevation in the density of the lung tissue, which conceals the underlying vessels and bronchial walls [30].

In interpreting the radio density of CT scan images quantitatively, radiologists use Hounsfield Units (HU), also known as CT units. The Hounsfield Unit (HU) threshold is a fundamental concept in medical imaging, particularly in the context of computed tomography (CT) scans. It serves as a quantitative measure used to differentiate between various types of tissues based on their radiodensity, as captured in CT images. Hounsfield units (HU) have a scale range larger than grayscale images in general, with 4096 intensity levels. HU values are derived from the linear attenuation coefficients of tissues and are calibrated based on the density of water (0 HU) and air (−1000 HU). The zero scale is assigned to distilled water at standard temperature and pressure, and air is given to the −1000 HU scale. The upper limit reaches about 1000 for bones, 2000 for denser bones such as the cochlea, and above 3000 for metals such as steel or silver. Tissue with higher X-ray absorption shows positive values and appears brighter, while tissue with lower X-ray absorption shows negative values and appears darker [31,38].

In clinical practice, the HU threshold is crucial for distinguishing between different anatomical structures and pathologies within the body. It allows for the categorization and segmentation of tissues, enabling radiologists and imaging software to identify specific areas of interest. For example, in lung imaging, the HU threshold is often utilized to differentiate between healthy lung tissue and pathologies such as pulmonary nodules, consolidations, or ground-glass opacities. Moreover, HU thresholds play a significant role in the quantitative assessment of tissue characteristics, aiding in the diagnosis and monitoring of various conditions. By setting specific HU ranges, it becomes possible to isolate and analyze regions of interest, which is vital in treatment planning, disease progression tracking, and overall patient management in clinical practice. The appropriate determination and utilization of HU thresholds contribute significantly to the accuracy and reliability of diagnostic interpretations in CT imaging. The physical thickness of tissue is directly related to the absorption of X-ray light.

### Dataset

In this study, we used chest CT scan data from 10 hospitals in Indonesia with suspect COVID-19 between June 2020 and September 2020 that has been tested using the reverse transcription-polymerase chain reaction (RT-PCR) for SARS-CoV-2 nucleic acid with nasopharyngeal or oropharyngeal swab specimens, which categorized of 11 patients positive, nine patients negative, six patients under Supervision (PUS), 14 patients under Investigation (PUI). The CT scan images have properties such as DICOM format with size 512×512, depth bit 16, and color type grayscale. The patient characteristics is shown in Table 1.

**Table 1:** Demographic and status patient characteristics.

Patient status	Patient characteristics	*n*	%
Negative		**9**	**22.5**
	Age (mean, range)	32.667 (8–47)	
	Age <40	3	33.3
	Age 40–68	6	66.7
	Age >68	0	0
	Male	5	56
Under Supervision (PUS)		**6**	**15**
	Age (mean, range)	36,83 (24–54)	
	Age <40	4	67
	Age 40–68	2	33
	Age >68	0	0
	Male	0	0.0
Under Investigation (PUI)		**14**	**35**
	Age (mean, range)	32,29 (2–49)	
	Age <40	9	67
	Age 40–68	5	33
	Age >68	0	0
	Male	7	50
Positive		**11**	**27.5**
	Age (mean, range)	56.54 (28–87)	
	Age <40	1	9
	Age 40–68	7	63
	Age >68	3	27
	Male	5	45

**Fig. 1:**
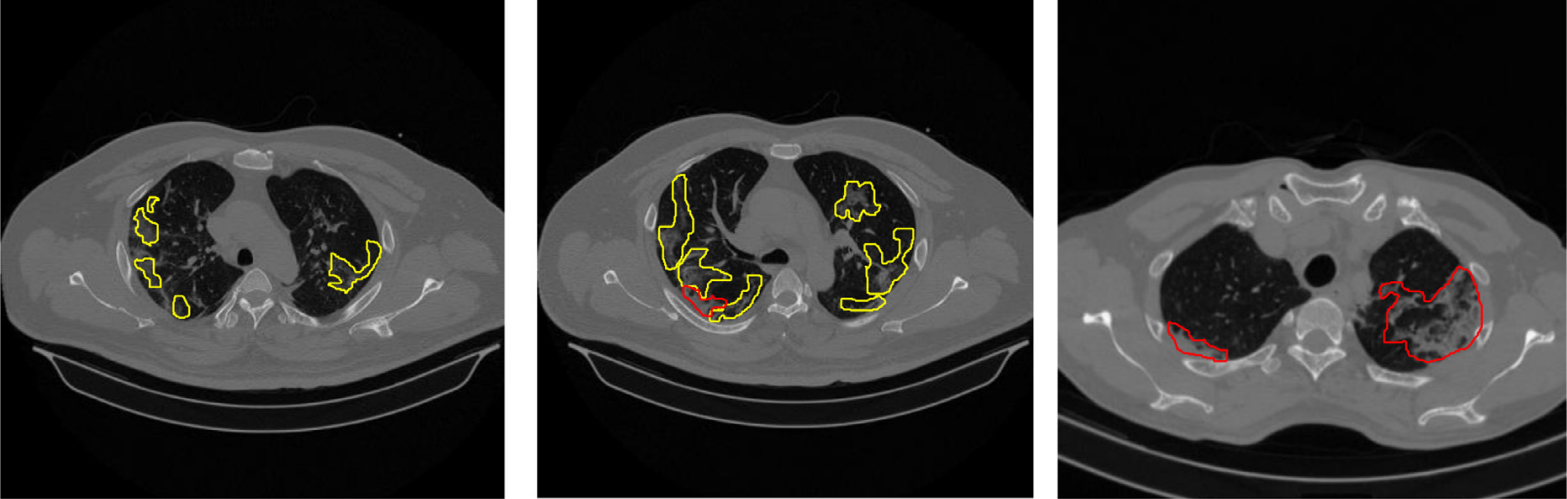
Several examples of annotated images (yellow: GGO, red: Consolidation).

Annotation is the process of labeling data based on medical assessments carried out by radiologists to be used as a reference value in measuring AI performance. The annotation process uses 3D Slicer, a tool that can provide labels as layer masks on medical images. 3D Slicer is also equipped with a tabulation feature to record the results of medical interpretations as tabulated data. Annotated CT scan images were selected from positive and under-surveillance patients. The number of annotation images is 12 slices consisting of 2 labels, namely GGO appearance and consolidation. Several examples of annotated images by radiologists are presented in Fig. 1, which is marked with yellow for GGO lesion areas and red for consolidation lesion areas.

### Imaging features detection method

**Fig. 2:**
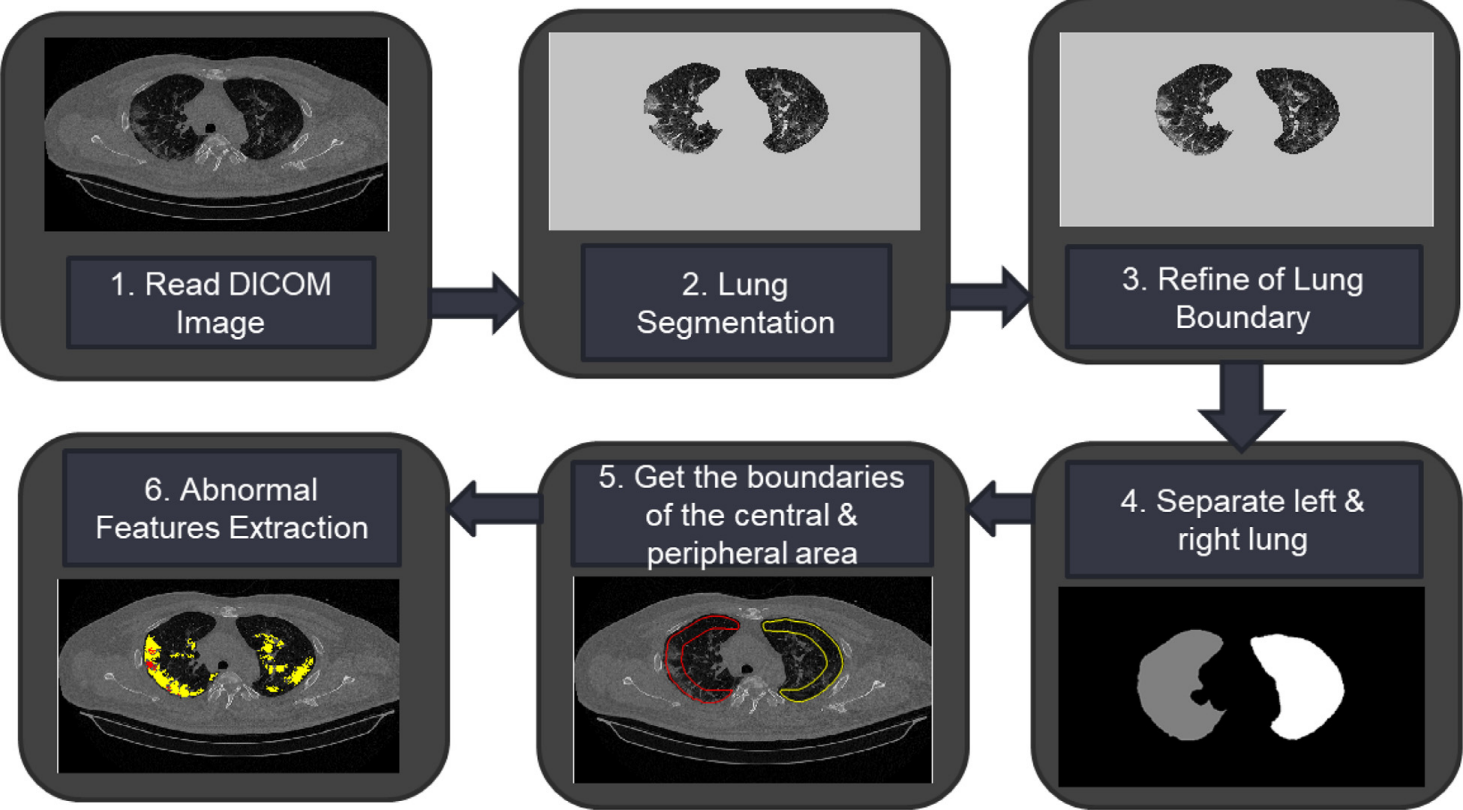
The proposed imaging features detection method*.*

**Fig. 3:**
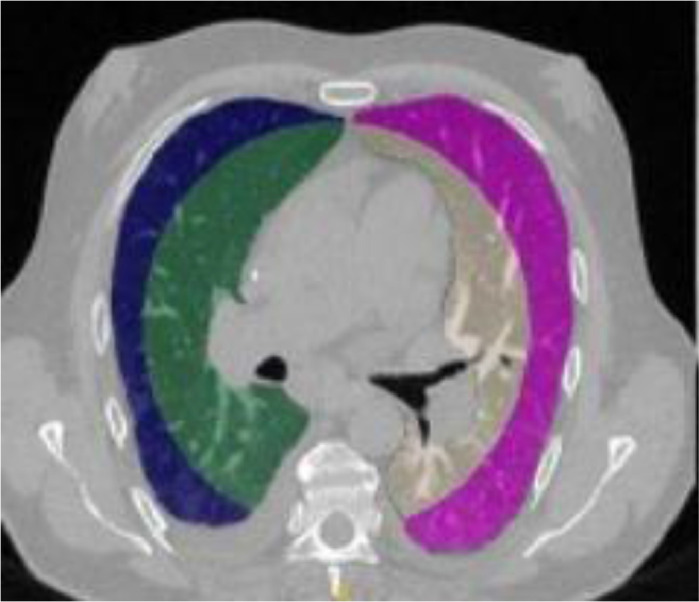
An example of central-peripheral division of lungs [39].

As shown in Fig. 2 diagram, the proposed method consists of several stages. The input image as a DICOM image is changed to type *int16*. Segmentation is performed to separate the lung field area from the background by using the HU threshold value and several morphological operations. The edges of the lung fields that are not well segmented due to opacity at the lung field boundaries need to be repaired using a convex hull on the lung fields. Simultaneously with the repair process, a separation process was also carried out between the left and right lung fields. The boundaries of the peripheral and central areas are determined for each left and right lung field. We need to identify the lung's peripheral area to determine the severity level because the location of the abnormality in the lungs also affects the assessment of COVID-19 or non-COVID-19. An example of dividing lung segmentation for the benefit of computer-assisted diagnosis shown in Fig. 3. The following process determines the GGO and Consolidation abnormality areas by using the HU threshold value in each part of the left peripheral, right peripheral, or left central and right central. The program code for the imaging features detection method can be accessed at: https://github.com/fajarastuti2008/ Detection-Method-of-Viral-Pneumonia.

**Fig. 4:**
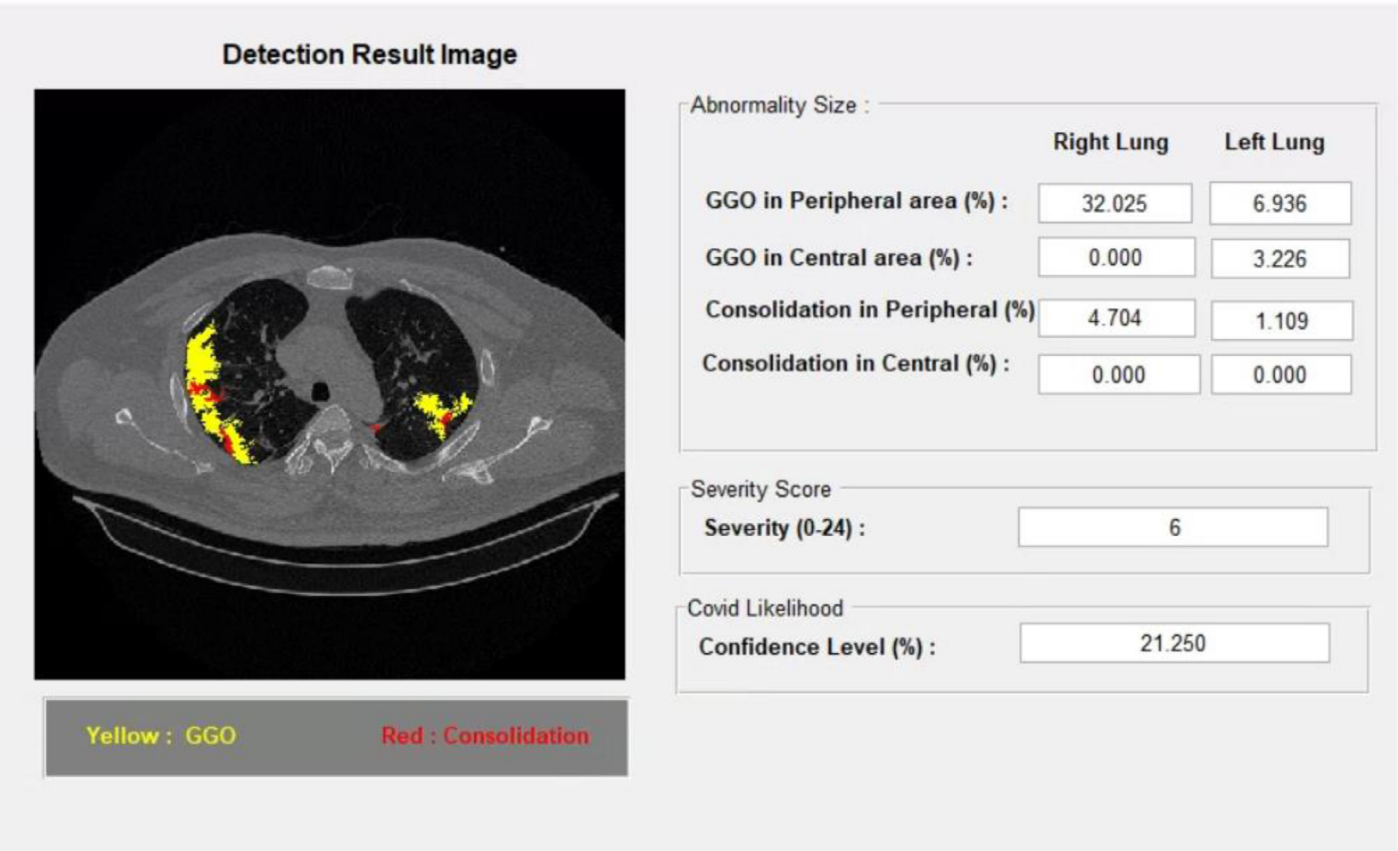
Sample result of the methods.

The output of this system displays lung field areas with abnormalities in the form of GGO and consolidation with different colors, as shown in Fig. 4. As well as showing the extent of the abnormality in percent in each area, namely right peripheral, left peripheral, right central, and left central. At the bottom is also displayed the severity score with a score between 0 and 24 and the level of confidence in the percentage indicating the level of confidence in the occurrence of COVID-19 on the reading of the CT scan image.

### Read DICOM image

This function aims to read the DICOM image and convert it into *int16* format to get the Hounsfield Unit (HU) scale. The function's input is the file name and location (path) of a single slice CT scan image in DICOM format (*I*). The function outputs a grayscale image with an HU scale in *int16* format (*I*_2_). This function consists of the following steps:

1. Reads the metadata of the appropriate DICOM file specified in filename
2. Reads image data (*I*) from messages referenced in the DICOM metadata structure
3. Convert image data (*I*) to *int16* format
4. Retrieve the *RescaleSlope* and *RescaleIntercept* values from the DICOM metadata structure
5. Obtain image data in HU scale (*I*_2_) with the Eq. (1).
  (1) $$I_{2}=RescaleSlope.I+RescaleIntercept$$

### Lung segmentation

**Fig. 5:**
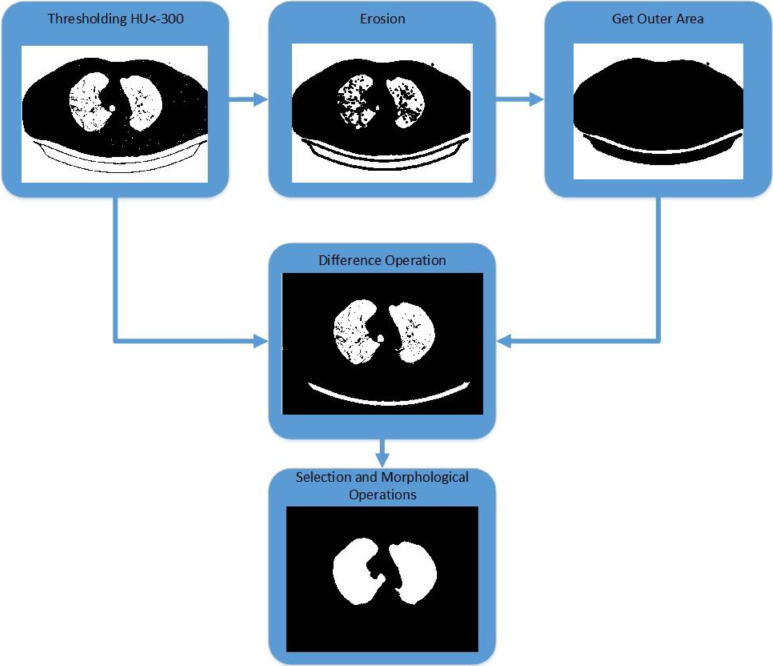
Lung segmentation steps.

The lung segmentation step aims to separates the respiratory organs (lung and airway) from other areas, which the input is a grayscale image with HU scale in *int16* format, *I*_2_. The function outputs the lung field images in binary format, *I_L_*. Thresholding techniques are used based on HU values and morphological operations in this stage. Let's assume that the input image from this process is *I*_2_(*x,y*), where *x,y* is an integer as a pixel coordinate, and *M*x*N* is size of the input image. The lung segmentation steps represent in Fig. 5 as follows:

1. Thresholding, a widely used method for segmenting regions [40], involves dividing an image into sets of pixels with values that are either lower or higher than the specified threshold. In this thresholding step, the grayscale image with HU scale is segmented to obtain the lung field area using a threshold value of Hounsfield Unit (HU) < −300. The output image of thresholding process, *I_th_*(*x,y*) is formulated as Eq. (2).
  (2) $$I_{th}\left(x,y\right)=\left\{ \begin{array}{cc} 1; & if\;I_{2}\left(x,y\right)<\;-300\\ 0; & otherwise \end{array}\right.$$
2. Erosion is one of the basic operations of morphological image processing to shrink foreground structures. This operation depends on the shape of the structuring elements [41]. This erosion step is used to separate areas connected by thin or small lines or curves. The erosion morphology operation implemented a structuring element 'sphere' with a diameter of 3. If *S*(*z,t*) is a structuring element with *z,t* is element coordinate, then using input *I_th_*(*x,y*), this process produces output *I_er_*(*x,y*) by Eq. (3) as in Ref. [42] :
  (3) $$I_{er}\left(x,y\right)=\min\left\{I_{th}\left(x+z,y+t\right)-S\left(z,t\right)\right\}$$
3. Get outer area: This step is used to get the external air area to be removed. By using the bounding box property of the each regions in the binary image from step 2, *I_er_*(*x,y*), select the bounding box area whose upper limit or left border is less than one or the lower limit is more than the row size of the image, or the right edge is more than the column size of the image. Consider the binary image, *I_er_*(*x,y*), have *k* regions, such as *R*_1_, *R*_2_, …, *R_k_*. If the bounding box of *R_i_*(*x,y*) has parameters *p, q, r, s*, where *p* is the leftmost column coordinate, and *q* is the top row coordinate. Meanwhile *r* and *s* are the width and height of the bounding, respectively, then the Eq. (4) determines the external air area, *I_ou_*(*x,y*).
  (4) $$I_{ou}\left(x,y\right)=\left\{ \begin{array}{cc} R_{i}\left(x,y\right); & if\;\left(p<1\right)|\left(p+r\right)\rangle \left(N-1\right)\left|\left(q<1\right)\right|\left(q+s\right)<\left(M-1\right))\\ 0; & otherwise \end{array}\right.$$
4. Difference Operation. It is a difference or subtraction operation between the thresholded image, $I_{th}\left(x,y\right),$ and the outer area image, $I_{ou}\left(x,y\right),$ which has been dilated four times with the same structuring element so that the exposed area of the external area image will be smaller. The dilation steps can be formulated by Eq. (5) as in Ref. [42].
  (5) $$I_{dil}\left(x,y\right)=\max\left\{I_{ou}\left(x-z,y-t\right)+S\left(z,t\right)\right\}$$

Next, the step dilation result, $I_{dil}\left(x,y\right)$, is operated using Eq. (6).

(6) $$I_{diff}\left(x,y\right)=I_{th}\left(x,y\right)-I_{dil}\left(x,y\right)$$

5. Selection and Morphological Operations. The area, which is the result of the subtraction operation, $I_{diff}\left(x,y\right),\;$is selected using the property
  ­ Area > 1000 pixels to get rid of small areas
  ­ Eccentricity < 0.9 to eliminate extended areas

Furthermore, closing morphological operator is applied to seal the small holes in the resulting lung field area. Closing is a dilation operation (Eq. (5)) followed by an erosion operation (Eq. (3)) using the same structuring element [42]. So, the result image in this step is the output image of the lung segmentation step, *I_L_*.

### Refine of lung boundary and separation of left and right lung fields

This step aims to repair the lung image boundaries and separate the left and right lung fields. The input of this step is a lung area in binary format, *I_L_*. Outputs are the refined lung area in binary form, *I_R_*, and the left and right lung area labels.

**Fig. 6:**
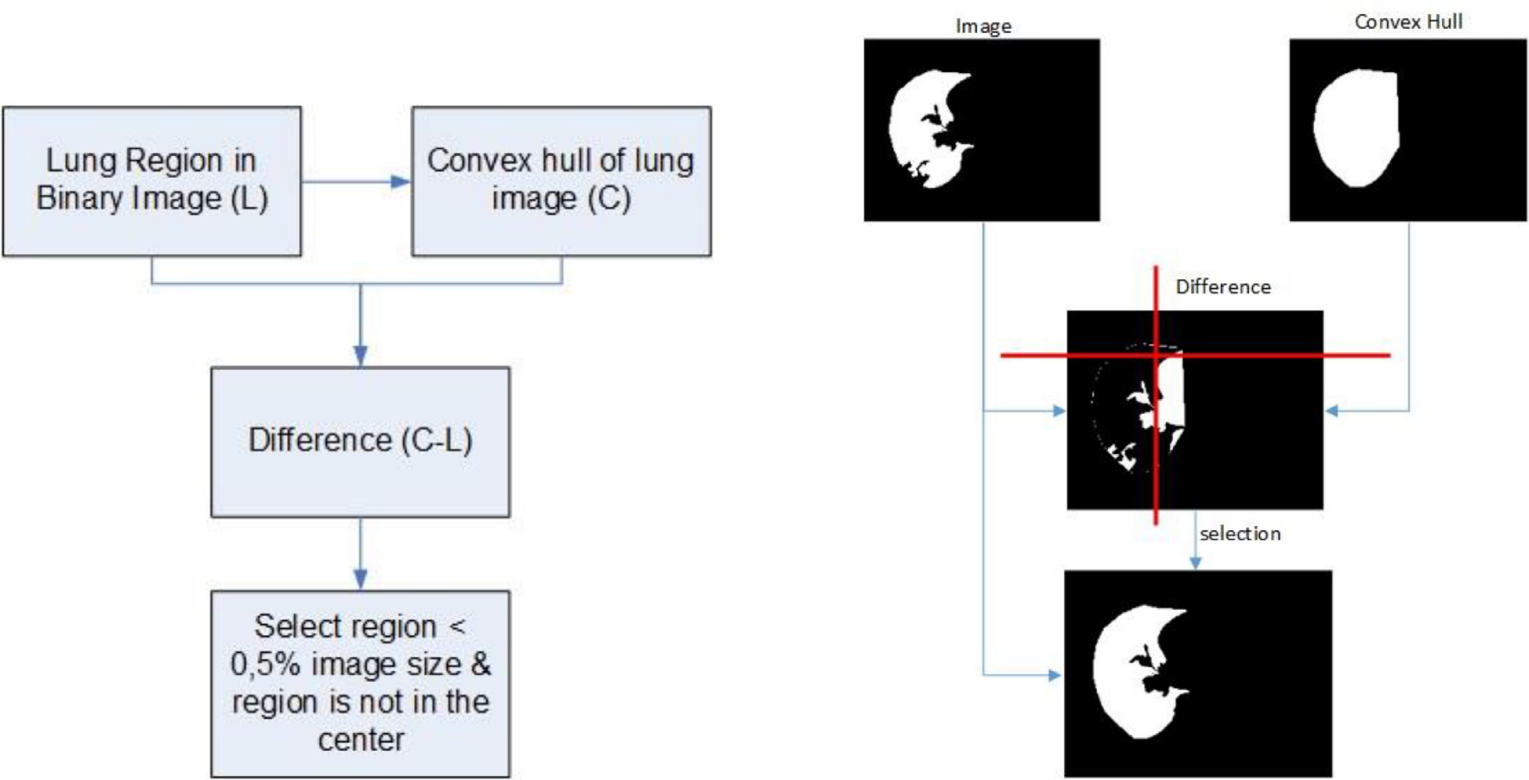
(left) Refine lung boundary steps (right) example of refine lung boundary steps.

Fig. 6 illustrates the refinement steps for delineating the lung boundary and provides an example. At this phase, the task involves correcting the boundaries of previously identified lung fields, which may have irregular shapes due to anomalies or significant intensity variations at their edges. This process aims to enhance the segmentation outcomes during the lung area extraction phase.

Initially, the approach adopts the convex hull algorithm as in Ref. [43], which determines the most minor convex shape, enclosing all the points within the lung region. Then, we calculate the difference between the total lung area (*I_L_*) and the convex hull of the lung area (*I_C_*). Subsequently, it selects an area that is less than 0.5% of the total image area and avoids segmentation within the central portion of the lung. Finally, the selected areas are added to the lung image (*I_L_*), as shown in Fig. 6 (right). The refining process is executed independently for each left and right lung field.

**Fig. 7:**
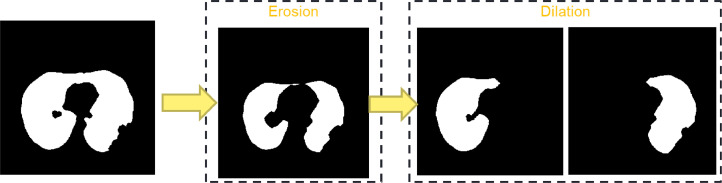
Refine steps for connected lung field.

If the lung region (*I_L_*) is connected, as shown in Fig. 7, an erosion morphology operation uses a size 3 “diamond” structuring element to separate the lung fields. The erosion process is repeated until two parts of the left and right lung fields are produced. After that, dilation was carried out with the same structuring elements as many erosion operations for each lung field.

### Obtain central and peripheral area

This function step obtains the peripheral and central areas of each left and right lung field. The inputs are (1) lung field images in binary format (*I_R_*), (2) label images of left and right lung fields, and (3) original images in grayscale (*I*_2_). This function generates six images as follows.

- Left lung field image in *int16* format.
- Right lung field image in *int16* format.
- Binary image of the left peripheral area.
- Binary image of the left central area.
- Binary image of the right peripheral area.
- Binary image of the right central area.

**Fig. 8:**
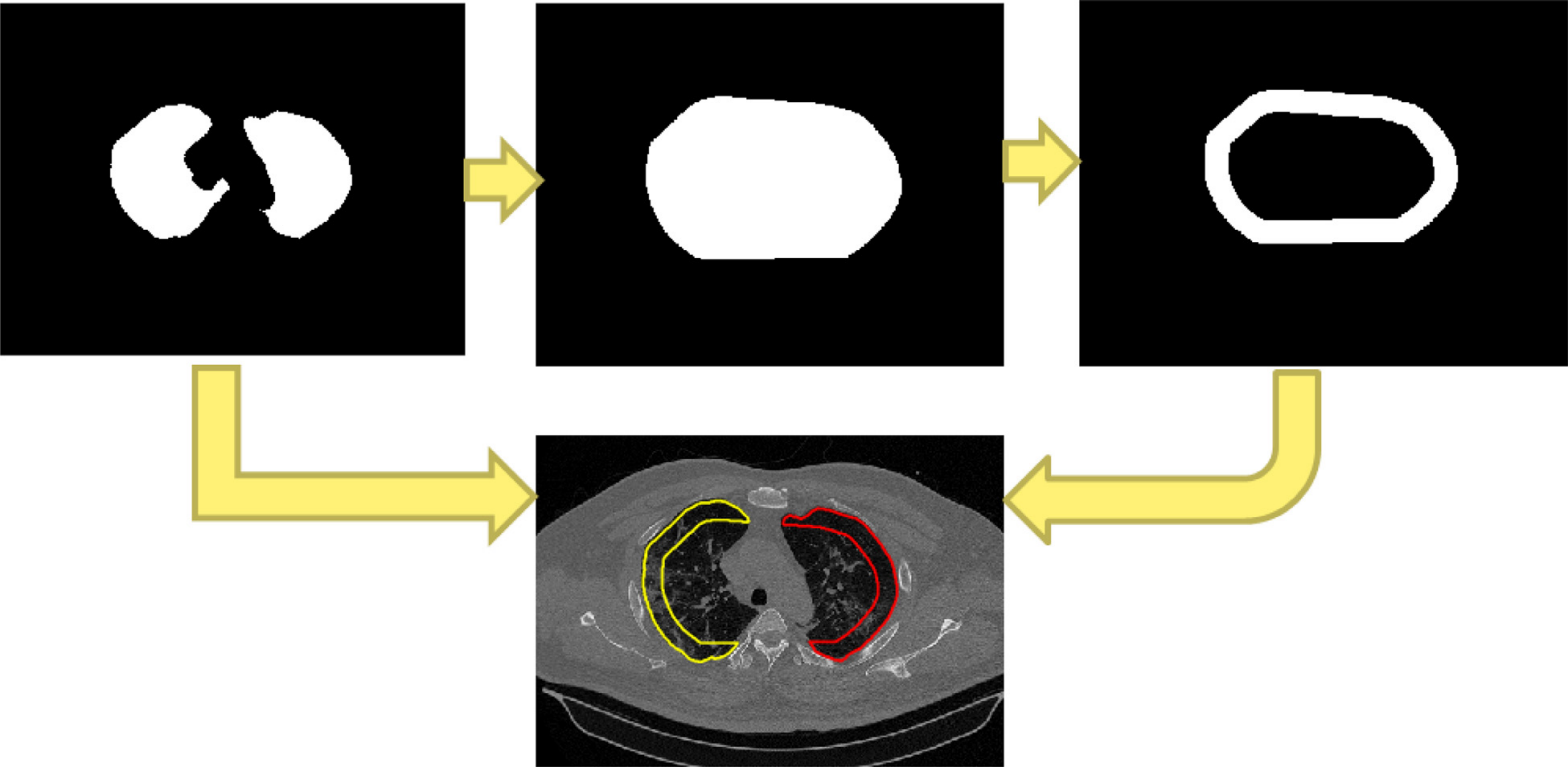
Obtain peripheral boundaries.

In this stage, depicted in Fig. 8, segmentation separates the areas into the left peripheral and left central regions in the lung fields, as well as the right peripheral and right central regions of the right lung fields. The approach involves deriving the convex hull area from a binary image of the lung fields. Subsequently, utilizing the *bwdist* function, the distance of all points or pixels within the convex hull area is calculated from the edge pixels. Pixels with a distance from the edge of less than 0.25 of the most significant distance are selected, defining the edge or peripheral area. Conversely, the area that exceeds 0.25 of the most significant distance constitutes the central area. Finally, the resulting peripheral and central regions are intersected with the lung field areas to derive the right peripheral, left peripheral, right central, and left central regions.

### Abnormal feature extraction

The function's purpose is to utilize HU scale information from segmented lung field images to extract GGO and consolidation features, specifically focusing on both peripheral and central regions. The required inputs include:

- An original image in grayscale format.
- Separate images of the left and right lung fields in HU format.
- Binary images representing the left and right peripheral areas.
- Binary images for the left and right central areas.

Upon execution, the function generates outputs that consist of:

- An RGB image derived from the original, showcasing GGO in yellow and consolidation in red.
- A feature vector sized 10×1, encompassing the feature area details of GGO and abnormalities detected in the analysis.

In this stage, a thresholding process is carried out to obtain GGO features with and consolidation using images at the HU scale where:

- GGO has a range of HU values: [−700, −300].
- Consolidation has a range of HU values: [−300, 50].

At this stage, the process of selecting and reducing areas that are not abnormal but have the same HU value as the abnormal area's HU value, such as blood vessels, is also carried out.

### Severity score and confidence level

The confidence score is calculated based on the extent of GGO and consolidation abnormalities in each peripheral and central area, as presented in Table 2. Table 3 shows the appearance level of abnormalities with a score of 4 for consolidation findings. The location score in Table 4 shows that the severity level in the peripheral region is higher than in the central area. In the example of a CT scan image, for example, having a GGO area in the left periphery of 35%, then the left peripheral GGO area score = 2, with an appearance score = 1 (GGO) and a location score = 3 in the periphery. The three scores were multiplied, so the left peripheral GGO score = 6. In the same way, the scores for each area were obtained. Then, the overall score is added and divided by the maximum score 160.

**Table 2:** Level/score for area of GGO/consolidation.

Area of GGO/Consolidation	Level/score
0	0
<25%	1
25–50%	2
50–75%	3
>75%	4

**Table 3:** Level/score for abnormalities appearance.

Appearance	Level/score
No finding	0
GGO	1
Consolidation	4

**Table 4:** Level/score for abnormalities location.

Location	Level/score
No finding	0
central	1
peripheral	3

Calculating the severity score of the appearance of abnormalities on CT scan images uses the calculation of the extent of GGO abnormalities and consolidation in the left and right lung fields in a manner that is almost the same as calculating the confidence level. For example, in the case of GGO in the left lung field of 36%, the area score = 2 and the appearance score = 1 (GGO), so the total GGO score in the left lung field = 2 × 1 = 2. In the same way, the extent of GGO abnormalities and consolidation in the right lung field were also determined. The total score of each lung field shows the severity level on the read CT scan image. This severity score is between 0 and 24.

## Method validation

**Fig. 9:**
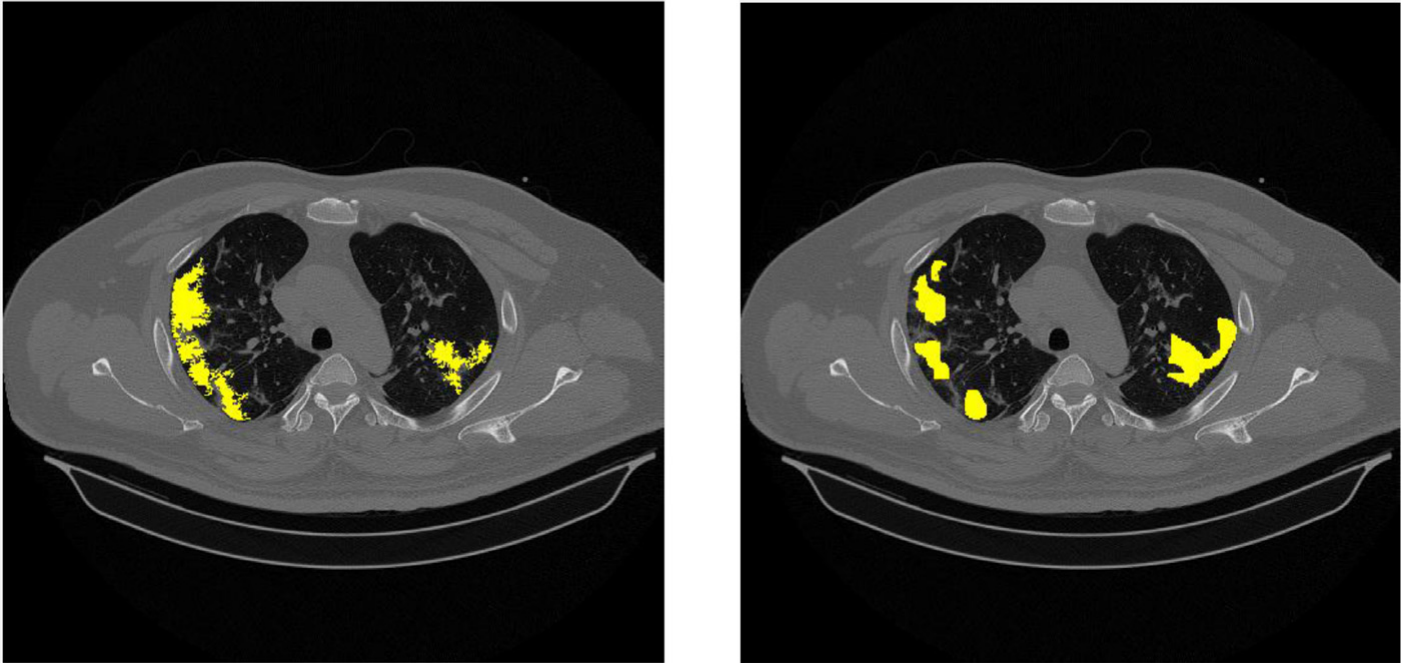
Comparison example of image id 34A (left) detection result (right) annotated image by radiologist.

**Fig. 10:**
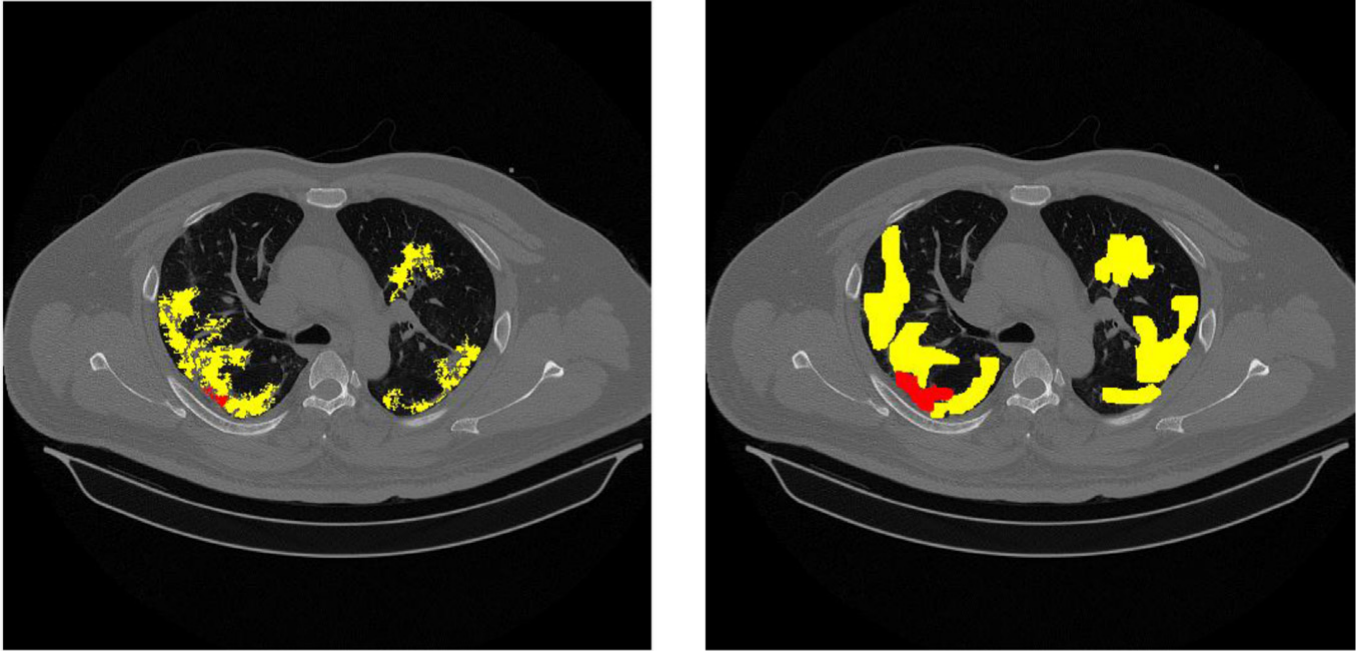
Comparison example of image id 34B (left) detection result (right) annotated image by radiologist.

**Fig. 11:**
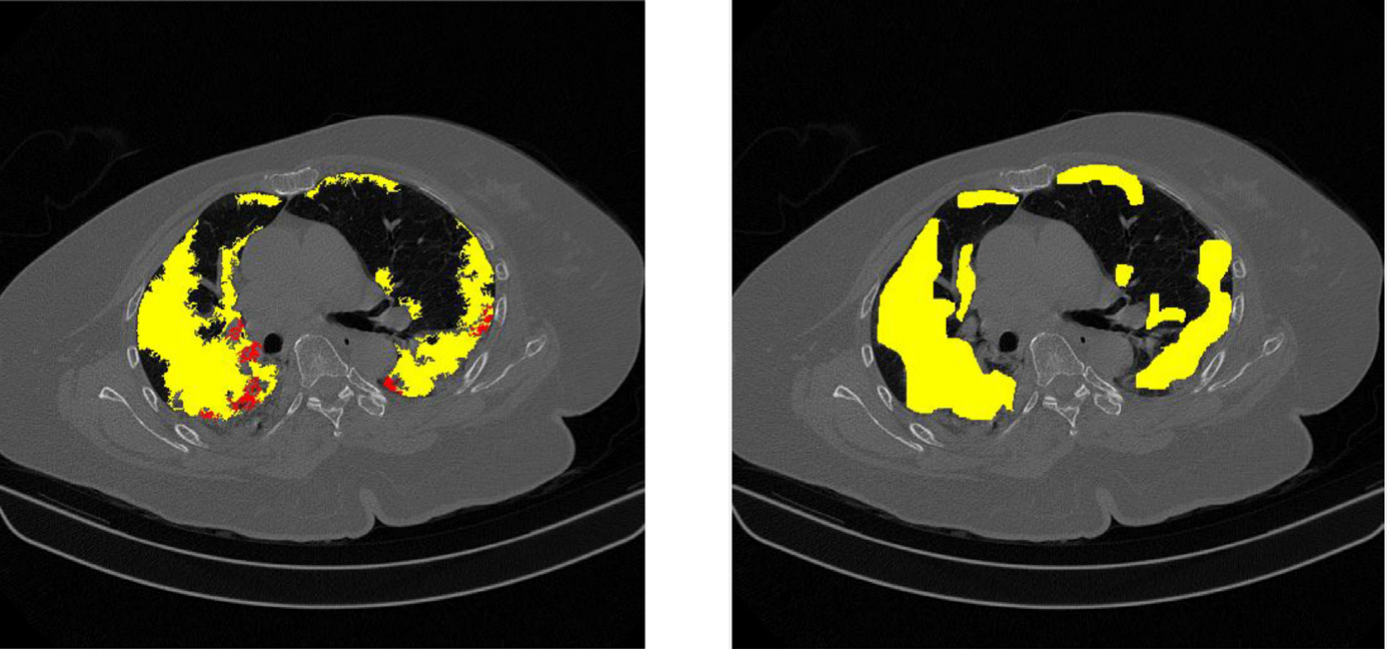
Comparison example of image id 36B (left) detection result (right) annotated image by radiologist.

To validate this method, we compare severity score calculation between some samples annotated images by radiologists and the computation result of this method. Table 5 shows the comparison, where the GT (Ground Truth) is the annotated image. From this table, the average error is 0,059 for the severity score and 0,069 for the confidence level. Fig. 9, Fig. 10, Fig. 11 visualize examples of the resulting and annotated images.

**Table 5:** Comparison of severity scores between annotated images and detection results.

No	Image Id	GGO in right peripheral	GGO in right central	GGO in left peripheral	GGO in left central	Consolidation in right peripheral	Consolidation in right central	Consolidation in left peripheral	Consolidation in left central	severity score	severity in percentage	confidence level
1	34A	0,320	0,000	0,069	0,032	0,000	0,000	0,000	0,000	2	0,083	0,063
	34A GT	0,233	0,000	0,146	0,040	0,000	0,000	0,000	0,000	2	0,083	0,044
	error	**0,087**	**0,000**	**0,077**	**0,008**	**0,000**	**0,000**	**0,000**	**0,000**	**0**	**0,000**	**0,019**
2	34 B	0,281	0,051	0,103	0,107	0,014	0,000	0,000	0,000	4	0,167	0,144
	34 B GT	0,407	0,095	0,303	0,226	0,080	0,000	0,000	0,000	6	0,250	0,163
	error	**0,126**	**0,045**	**0,200**	**0,119**	**0,066**	**0,000**	**0,000**	**0,000**	**2**	**0,083**	**0,019**
3	34C	0,135	0,002	0,105	0,000	0,007	0,000	0,000	0,000	4	0,167	0,119
	34C GT	0,145	0,000	0,183	0,000	0,000	0,000	0,000	0,000	2	0,083	0,038
	error	**0,010**	**0,002**	**0,077**	**0,000**	**0,007**	**0,000**	**0,000**	**0,000**	**2**	**0,083**	**0,081**
4	34D	0,200	0,033	0,121	0,018	0,007	0,000	0,008	0,000	6	0,250	0,200
	34D GT	0,217	0,020	0,319	0,109	0,000	0,000	0,000	0,000	3	0,125	0,069
	error	**0,017**	**0,013**	**0,198**	**0,090**	**0,007**	**0,000**	**0,008**	**0,000**	**3**	0,125	**0,131**
5	34E	0,153	0,000	0,066	0,000	0,014	0,018	0,000	0,000	4	0,167	0,138
	34E GT	0,398	0,227	0,320	0,160	0,000	0,000	0,000	0,000	4	0,167	0,088
	error	**0,245**	**0,227**	**0,254**	**0,160**	**0,014**	**0,018**	**0,000**	**0,000**	**0**	0,000	**0,050**
6	34F	0,137	0,086	0,139	0,000	0,048	0,000	0,013	0,000	6	0,250	0,194
	34F GT	0,342	0,491	0,325	0,000	0,000	0,000	0,000	0,000	3	0,125	0,088
	error	**0,205**	**0,405**	**0,186**	**0,000**	**0,048**	**0,000**	**0,013**	**0,000**	**3**	**0,125**	**0,106**
7	36A	0,602	0,388	0,354	0,008	0,086	0,043	0,061	0,012	8	0,333	0,313
	36A GT	0,791	0,531	0,541	0,018	0,000	0,000	0,000	0,000	5	0,208	0,156
	error	**0,189**	**0,143**	**0,187**	**0,010**	**0,086**	**0,043**	**0,061**	**0,012**	**3**	0,125	**0,156**
8	36B	0,567	0,519	0,410	0,080	0,034	0,051	0,026	0,000	9	0,375	0,294
	36B GT	0,661	0,539	0,467	0,123	0,000	0,000	0,000	0,000	5	0,208	0,119
	error	**0,094**	**0,020**	**0,057**	**0,043**	**0,034**	**0,051**	**0,026**	**0,000**	**4**	**0,167**	**0,175**
9	36C	0,487	0,288	0,442	0,102	0,043	0,000	0,044	0,024	8	0,333	0,269
	36C GT	0,489	0,191	0,363	0,083	0,199	0,071	0,166	0,026	8	0,333	0,288
	error	**0,002**	**0,097**	**0,080**	**0,019**	**0,157**	**0,071**	**0,122**	**0,002**	**0**	**0,000**	**0,019**
10	36D	0,375	0,209	0,402	0,071	0,048	0,015	0,073	0,000	8	0,333	0,263
	36D GT	0,588	0,166	0,409	0,006	0,096	0,032	0,029	0,000	8	0,333	0,281
	error	**0,213**	**0,043**	**0,007**	**0,064**	**0,049**	**0,017**	**0,044**	**0,000**	**0**	**0,000**	**0,019**
11	36E	0,439	0,139	0,397	0,294	0,056	0,000	0,040	0,000	8	0,333	0,244
	36E GT	0,513	0,222	0,401	0,063	0,192	0,033	0,085	0,000	8	0,333	0,281
	error	**0,074**	**0,083**	**0,005**	**0,231**	**0,136**	**0,033**	**0,044**	**0,000**	**0**	**0,000**	**0,038**
12	36F	0,511	0,504	0,495	0,772	0,031	0,000	0,079	0,000	10	0,417	0,288
	36F GT	0,627	0,725	0,583	0,869	0,021	0,000	0,037	0,000	10	0,417	0,306
	error	**0,116**	**0,221**	**0,088**	**0,096**	**0,010**	**0,000**	**0,042**	**0,000**	**0**	**0,000**	**0,019**
**Average**		**0,115**	**0,108**	**0,118**	**0,070**	**0,051**	**0,019**	**0,030**	**0,001**		**0,059**	**0,069**

As depicted in Table 5, for image Id 34A, the detection results indicate the presence of GGO appearance in the left peripheral area covering 32%, 6.9% in the right peripheral, and 3.2% in the right central region. Based on the calculations from Table 2, Table 3, Table 4, a severity score of 2 was obtained with a confidence level of 6.3%. The severity score calculation for the ground truth (GT) image also shows the same number. This is supported by the visualization in Fig. 9. The same thing can be observed in Figs. 10 and 11 for image IDs 34B and 36B. This indicates that the results from the feature detection method offered yield outcomes that are not significantly different from our radiologists' assessments.

## Ethics statements

The human participatory studies were reviewed and approved by the Ethics Committee of the Dr. Cipto Mangunkusumo National Central Public Hospital in 2020. This study did not require written consent to participate in accordance with national law and institutional requirements. Written permission was waived as this was a retrospective study with no potential risk to the patient.

## CRediT authorship contribution statement

**Fajar Astuti Hermawati:** Conceptualization, Methodology, Software. **Bambang Riyanto Trilaksono:** Project administration. **Anto Satriyo Nugroho:** Methodology. **Elly Matul Imah:** Methodology. **Lukas:** Methodology. **Telly Kamelia:** Methodology, Validation. **Tati L.E.R. Mengko:** Methodology. **Astri Handayani:** Methodology. **Stefanus Eric Sugijono:** Data curation, Validation. **Benny Zulkarnaien:** Data curation, Validation. **Rahmi Afifi:** Data curation, Validation. **Dimas Bintang Kusumawardhana:** Resources.

## Declaration of Competing Interest

The authors declare that they have no known competing financial interests or personal relationships that could have appeared to influence the work reported in this paper.

## Data Availability

Data will be made available on request.
